# Playing on the Dark Side: SMYD3 Acts as a Cancer Genome Keeper in Gastrointestinal Malignancies

**DOI:** 10.3390/cancers13174427

**Published:** 2021-09-02

**Authors:** Paola Sanese, Candida Fasano, Cristiano Simone

**Affiliations:** 1 Medical Genetics, National Institute for Gastroenterology, IRCCS “S. de Bellis” Research Hospital, 70013 Castellana Grotte, Italy; paola.sanese@irccsdebellis.it (P.S.); candida.fasano@irccsdebellis.it (C.F.); 2 Medical Genetics, Department of Biomedical Sciences and Human Oncology (DIMO), University of Bari Aldo Moro, 70124 Bari, Italy

**Keywords:** SMYD3, gastrointestinal tumors, DNA damage response, cell cycle checkpoints, homologous recombination repair, synthetic lethality, SMYD3 inhibition

## Abstract

**Simple Summary:**

The activity of SMYD3 in promoting carcinogenesis is currently under debate. Growing evidence seems to confirm that SMYD3 overexpression correlates with poor prognosis, cancer growth and invasion, especially in gastrointestinal tumors. In this review, we dissect the emerging role played by SMYD3 in the regulation of cell cycle and DNA damage response by promoting homologous recombination (HR) repair and hence cancer cell genomic stability. Considering the crucial role of PARP1 in other DNA repair mechanisms, we also discuss a recently evaluated synthetic lethality approach based on the combined use of SMYD3 and PARP inhibitors. Interestingly, a significant proportion of HR-proficient gastrointestinal tumors expressing high levels of SMYD3 from the PanCanAtlas dataset seem to be eligible for this innovative strategy. This promising approach could be taken advantage of for therapeutic applications of SMYD3 inhibitors in cancer treatment.

**Abstract:**

The SMYD3 methyltransferase has been found overexpressed in several types of cancers of the gastrointestinal (GI) tract. While high levels of SMYD3 have been positively correlated with cancer progression in cellular and advanced mice models, suggesting it as a potential risk and prognosis factor, its activity seems dispensable for autonomous in vitro cancer cell proliferation. Here, we present an in-depth analysis of SMYD3 functional role in the regulation of GI cancer progression. We first describe the oncogenic activity of SMYD3 as a transcriptional activator of genes involved in tumorigenesis, cancer development and transformation and as a co-regulator of key cancer-related pathways. Then, we dissect its role in orchestrating cell cycle regulation and DNA damage response (DDR) to genotoxic stress by promoting homologous recombination (HR) repair, thereby sustaining cancer cell genomic stability and tumor progression. Based on this evidence and on the involvement of PARP1 in other DDR mechanisms, we also outline a synthetic lethality approach consisting of the combined use of SMYD3 and PARP inhibitors, which recently showed promising therapeutic potential in HR-proficient GI tumors expressing high levels of SMYD3. Overall, these findings identify SMYD3 as a promising target for drug discovery.

## 1. Introduction

SMYD3 is a member of the SMYD (SET and MYND Domain) lysine methyltransferase family, which includes five members (SMYD1–5) [1]. Their methyltransferase activity requires the combination of the SET domain with adjacent cysteine-rich regions, one located N-terminally (pre-SET or N-SET) and the other posterior to the SET domain (post-SET). Pre- and post-SET domains seem to play a crucial role in the substrate recognition and enzymatic activity of SMYD family members [1,2]. The MYND domain is the structural discriminant between SMYDs and other SET domain-containing proteins and is found in several transcriptional regulators, in which it facilitates the interactions with partner proteins through PXLXP motifs [3]. Structural analyses showed that the N-terminal region of human SMYD3 includes the SET, MYND and post-SET domains, while the C-terminal region contains a tetratricopeptide repeat (TPR)-like domain that modulates SMYD3 interaction with the consensus motif MEEVD of HSP90 and other proteins, and its nuclear localization [1,4,5].

SMYD3 was first characterized as a histone H3 lysine 4 (H3K4) methyltransferase, with studies carried out in SMYD3-knocked down cell cultures confirming that its genetic ablation was often associated with reduced H3K4 methylation [6,7,8,9]. On the other hand, Van Aller and colleagues demonstrated that the preferred target of SMYD3-mediated methylation in vitro is H4K5 [10], which is consistent with results from our group [9].

Subsequent studies revealed that SMYD3 is an important epigenetic regulator that acts in both the nuclear and the cytoplasmic compartments and can interact with and methylate both histone and non-histone proteins. At the nuclear level, SMYD3 was initially shown to be recruited at CCTCCC DNA sequences [6]. Subsequently, genome-wide approaches revealed that it binds to DNA at target regions of transcription factors involved in cell proliferation [11]. Furthermore, SMYD3 is a crucial member of the transcriptional complex formed by RNA polymerase II and the RNA helicase HELZ [6] and serves as a coactivator of transcription processes [12,13,14]. In the cytoplasm, SMYD3 has been found to affect key factors involved in oncogenic pathways by interacting with and methylating non-histone proteins, which suggests a role as a modulator of signaling cascades promoting tumor progression [15,16,17,18].

SMYD3 activity does not appear to be required for normal development, as demonstrated by recent studies in SMYD3 knockout (KO) mice [11,15]. These results were confirmed in male and female SMYD3 homozygous conditional KO mice, which did not show significant abnormalities after whole phenotyping [19]. It has been reported that SMYD3 overexpression in normal cells is sufficient to accelerate cell growth and trigger the activation of genes involved in the transformation and migration of cancer cells [7,8]. Consistently, a close correlation has been observed between SMYD3 activity and the development of several malignancies. Indeed, SMYD3 has been found overexpressed in several types of cancers, including colorectal (CRC), breast (BC), gastric (GC), pancreatic (PC), ovarian (OvCa), prostate and lung cancer, and hepatocellular carcinoma (HCC) [1], with high SMYD3 levels being associated with reduced overall survival and worse prognosis [11,20,21,22,23,24,25].

Recent studies provided evidence that SMYD3 may be an important biomarker for the diagnosis of several types of cancers and a potential target for drug discovery [1]. As a result, several SMYD3 chemical compounds able to inhibit its enzymatic activity were recently developed [26]. In 2015, we identified the first substrate competitive SMYD3 inhibitor (SMYD3i), named BCI-121, by virtual screening. This compound showed antigrowth properties and confirmed the potential of targeting this protein [9]. Then, three more potent reversible SMYD3is (EPZ031686, EPZ030456 and, later, EPZ028862) were developed by the biopharmaceutical company Epizyme. These compounds have nanomolar potency and are therefore amenable to be used in in vivo assays [27,28]. Another SMYD3i described in the literature, named GSK2807, acts as a competitive ligand at the cofactor binding site [29]. Moreover, the existing drug Diperodon has been reported as a new allosteric ligand interacting with SMYD3 and represents a good starting point for the design of compounds acting as modulators of noncatalytic SMYD3 functions [30]. In addition to these, recent studies identified new selective SMYD3is (e.g., BAY-6530, covalent inhibitors 1–4) [26,31], thereby contributing to the ongoing efforts to develop effective SMYD3is that might be used as anticancer drugs.

The following sections provide a molecular picture of SMYD3 oncogenic functions, focusing on the gastrointestinal (GI) compartment, in which a particularly important role has emerged for this protein. Indeed, recent studies shed light on how SMYD3 affects GI cancer progression and promotes oncogenic features. In detail, we will discuss SMYD3 activity in orchestrating DNA damage checkpoint dynamics, driving cell cycle phase transition, and supporting genomic protection of cancer cells. Moreover, we will review recent data on the combined use of SMYD3i and PARP inhibitor (PARPi) as a synthetic lethality approach in GI cancers showing HR proficiency and high SMYD3 expression.

## 2. SMYD3 Oncogenic Functions

### 2.1. SMYD3 Exerts Oncogenic Effects through Multiple Mechanisms

Growing evidence supports a key role for SMYD3 in tumorigenesis in several cancer types. SMYD3 oncogenic activity has been linked to proliferation, cell cycle regulation, increased migration and invasion of cancer cells through multiple mechanisms, including chromatin remodeling, regulation of gene expression and interaction with and methylation of non-histone proteins.

**Figure 1:**
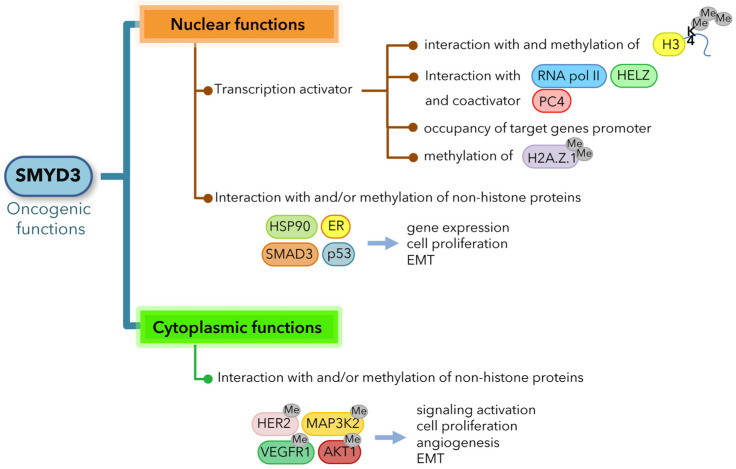
SMYD3 acts as an activator of multiple oncogenic mechanisms, which can be classified based on the cellular compartment in which they occur. RNA Pol II: RNA polymerase II.

SMYD3 has been found to exert its oncogenic effects through transcriptional activation of a set of downstream target genes involved in cell death and proliferation (e.g., *hTERT*, *WNT10B*) [32,33], epithelial-mesenchymal transition (EMT) (e.g., *SLUG*, *MMP2*, *MET*) [11,20,21,34,35,36], cell cycle regulation (e.g., *CCNA1*, *CCNA2*, *CCND1*, *CCNE1*, *PCNA*, *CDK2*) [11,14,21,37], stem cell maintenance (e.g., *ASCL2*) [38], as well as oncogenes (e.g., *MYC*, *JAK1/2*, *CTNNB1*) [11]. Several studies investigated the mechanisms by which SMYD3 promotes the transcription of target genes, showing that it can directly occupy their promoter regions, interact with the transcriptional machinery, form complexes with RNA polymerase II [6,11] and other coactivators, such as PC4 [13], and associate with active chromatin by interacting with H3K4me3 tails [11]. Furthermore, it has been reported that SMYD3 dimethylates and colocalizes with the histone variant H2A.Z.1 at the promoter of the *CCNA1* gene, inducing its expression and G1/S progression [14] (Figure 1).

In addition to regulating gene expression, SMYD3 has been shown to play a significant role in human cancer by modulating various key cancer-associated factors and therefore their related oncogenic pathways. Intriguingly, several studies revealed that SMYD3 exerts its oncogenic role primarily by interacting with and methylating non-histone proteins, through which it transactivates specific pathways involved in the survival and expansion of cancer cells [5,12,15,16,17]. In lung cancers and PCs, SMYD3 has a pivotal role in the regulation of oncogenic RAS signaling through the methylation of MAP3K2 kinase on lysine 206, which induces MAP3K2 release from the negative regulator PP2A phosphatase complex and therefore promotes ERK1/2 phosphoactivation [15]. Consistent with these findings, SMYD3 deletion or pharmacological inhibition resulted in lower ERK1/2 phosphorylation and thus reduced MEK-ERK signaling and tumor development in response to oncogenic RAS in CRCs and PCs [9,15]. In addition, SMYD3 can methylate lysine 14 on the AKT1 kinase, which promotes its phosphoactivation and plasma membrane accumulation, suggesting that SMYD3 methyltransferase activity may trigger the constitutive activation of AKT1 in cancer cells [16]. SMYD3 was also reported to interact with the estrogen receptor (ER) and potentiate ER-driven transcription, thereby promoting ER-mediated tumorigenicity [12]. It has been further shown that SMYD3 interaction with p53, which promotes p53 translocation into the cytoplasm and subsequent degradation, and its association with SMAD3 are both involved in mechanisms that mediate EMT [34,39]. Moreover, SMYD3 methylates two different receptor tyrosine kinases: vascular endothelial growth factor receptor 1 (VEGFR1), thereby potentiating angiogenesis through ligand-dependent autophosphorylation and increasing VEGFR1 kinase activity [17], and human epidermal growth factor receptor 2 (HER2), thereby enhancing HER2 homodimerization and subsequent autophosphorylation [18] (Figure 1).

Overall, SMYD3 is a versatile coregulator of multiple oncogenic pathways, affecting processes associated with gene expression and protein transactivation through which it integrates cellular signals and promotes cancer development (Figure 1).

### 2.2. SMYD3 and Cancer Cell Growth: An Emerging Debate

Several lines of evidence support the hypothesis that SMYD3 upregulation has a key role in tumorigenesis and cancer development in a number of human malignancies. Most of the studies performed to date showed a correlation between SMYD3 overexpression and cell growth in cancer settings. Knockdown of SMYD3 has been reported to decrease cell proliferation in a wide variety of cancers [6,7,10,15,22,23,32,35,36,40,41,42], while its overexpression promotes cell growth, transformation and reduces apoptosis sensitivity [6,43]. Based on these findings, small-molecule SMYD3is have been generated, and several studies showed that SMYD3 inhibition affects cellular proliferation [9,27,29].

However, a recent paper by Thomenius et al. has called into question the role of SMYD3 in cancer cell growth by showing that SMYD3i or SMYD3 KO with the novel CRISPR/Cas9 technology failed to impair cell proliferation of hundreds of cancer cell lines of different origin and genetic background. Based on these findings, the authors concluded that SMYD3 is not required for autonomous proliferation of cancer cells in vitro [28]. This result also highlighted that the identification of new high-quality compounds that can inhibit SMYD3, as well as the proper use of gene editing approaches allowing to overcome artifacts associated with RNAi technology, may help clarify the complex role played by SMYD3 in cancer biology.

In vivo studies on mice models seem to support SMYD3 involvement in tumorigenesis [9,11,15]. In a previous paper by our group, the expression and activity of SMYD3 were evaluated in a preclinical model of CRC, i.e., APC^Min/+^ mice treated with the carcinogen azoxymethane (AOM), and found to be strongly upregulated throughout tumorigenesis at both the mRNA and the protein levels, along with its downstream targets [9]. In another report, Mazur et al. showed that SMYD3 deficiency inhibits tumor development in mouse models of pancreatic ductal adenocarcinoma and lung adenocarcinoma, demonstrating that SMYD3 activity promotes the formation of RAS-driven carcinomas [15]. In line with these data, it has been shown that SMYD3 is required in mice for the development of chemically induced liver and colon carcinogenesis [11].

Altogether, in vivo studies and the above-described sophisticated work carried out by Thomenius et al. in cellular models [28] suggest that investigating the role of SMYD3 in tumorigenesis and cancer progression requires representative and reliable model systems allowing to preserve cellular heterogeneity and obtain biochemical and morphological characteristics that are similar to in vivo cancer tissue, as they influence gene expression and cell behavior [44]. In this light, in-depth studies of the functional role of SMYD3 and its overexpression in cancer are instrumental to elucidate the mechanism by which it regulates oncogenic progression.

## 3. SMYD3 Alterations in GI Cancers

In a recent report, we evaluated *SMYD3* somatic alterations and mRNA expression in CRCs and PCs by analyzing publicly available human cancer data from The Cancer Genome Atlas (TCGA) dataset, which contains records for many cancer types, including multi-omics and standardized clinical information. Our study revealed that only a very low percentage of these types of tumors harbored *SMYD3* alterations, including missense, fusion or deleterious mutations (around 1%), and a small fraction displayed copy number amplifications [45]. Specifically, among 459 CRC tumors, six cases showed copy number variations, including two deletions and four amplifications. Only eight tumors carried single nucleotide variations involving the *SMYD3* gene, including four missense mutations, three truncating mutations and only one predicted splicing variant. Similarly, among 157 PC tumors, our analysis revealed only one deleterious mutation. Conversely, SMYD3 mRNA levels were found increased in 29% and 27% of CRC and PC tumors, respectively [45].

This finding is in agreement with previous reports on cancer tissues and cell lines, suggesting that SMYD3 overexpression is an important player in the pathogenesis of GI cancers. As previously mentioned, SMYD3 has been found highly overexpressed in several types of cancers [1], with a preponderance of GI malignancies, in which SMYD3 overexpression correlates with poor prognosis and promotes cancer growth, migration and invasion. Hamamoto et al. initially characterized SMYD3 overexpression and its oncogenic functions in HCC and CRC [6]; subsequently, several groups demonstrated that upregulated SMYD3 promotes tumorigenicity and metastasis in various GI cancer types. In a previous paper, we reported that RNA interference (RNAi)-mediated SMYD3 ablation impairs CRC cell proliferation, suggesting that SMYD3 is required for proper cancer cell growth. Extended analysis revealed that SMYD3 is overexpressed in a number of GI cancer cell lines of different origins, with cells expressing high levels of SMYD3 being highly sensitive to its genetic depletion or pharmacological inhibition [9]. In addition, SMYD3 overexpression was associated with advanced T stage and lower survival rates in patients with CRC, thereby identifying it as an independent prognostic factor for this type of cancer [46]. Analysis of SMYD3 mRNA expression in HCC and adjacent non-tumor liver tissues showed that it is upregulated in HCC tumor tissues compared to paired normal tissues [21], with overexpression positively correlating with invasion, poor tumor differentiation, high TNM stage and a poor prognosis for patients with early-stage disease [20,21]. SMYD3 levels in HCC were also analyzed in TCGA dataset, confirming that its upregulation is correlated with the development of new tumor foci, HCC progression to high-grade disease and poorer overall survival [11]. SMYD3 overexpression has been detected in GC tissues and recognized as possible risk and prognostic factor in GC patients [47]. High levels of SMYD3 have also been positively correlated with STAT3 [48], MMP-9 [49] and TGFb1 [50] expression, and reported to repress the anticancer effects of sulforaphane [51]. Moreover, increased expression of SMYD3 has been found in esophageal squamous cell carcinoma, where it is associated with lymph node metastasis and poor overall survival [22,23,24]. In pancreatic adenocarcinoma, high SMYD3 levels positively correlated with tumor size, TNM stage, perineural invasion, lymph node metastasis and shorter survival [25]. Remarkably, SMYD3 was found overexpressed in RAS-driven PC, and its genetic ablation inhibited the spontaneous formation of pancreatic intraepithelial neoplasia caused by induced *KRAS* mutations and extended the lifespan of affected mice [15].

Collectively, the numerous studies reporting a positive correlation between high SMYD3 levels and GI cancer progression suggest that its upregulation is a potential risk factor in the biological behavior and prognosis of GI malignancies.

## 4. Oncogenic Role of SMYD3 in GI Cancers

### 4.1. Role of SMYD3 in Controlling Cell Cycle Progression

Over the past decade, most of the reports aimed at investigating SMYD3 function in cancer focused on the effect of its overexpression on cell cycle progression. As previously described, many of these studies revealed that SMYD3 influences cancer cell proliferation [1], showing that it is involved in controlling how the cell cycle clock ticks. It has been reported that overexpressed SMYD3 regulates cell growth by causing an acceleration of cancer cell division through modulation of the cell cycle [9,14,37,42,52]. Previous work by our group showed that SMYD3 affects cell cycle progression, revealing that its pharmacological inhibition by the novel small-molecule compound BCI-121 effectively reduces CRC cell proliferation by arresting cell cycle at the S/G2 boundary. This suggests the potential involvement of SMYD3 in the S/G2 checkpoint and hence in cell cycle deregulation, one of the critical steps in cancer development [9]. How SMYD3 affects cell cycle checkpoints is currently under study. Due to its ability to modulate chromatin accessibility, it can promote the transcription of several cell cycle-related genes. Sarris et al. demonstrated that SMYD3 occupies regulatory regions of genes involved in cell cycle control, such as *CCNA2*, *CCNE1*, *CCND1*, *PCNA*, *IGFBP1*, *MYC* and *CTNNB1*, and showed that their expression decreases in the liver and colon of carcinogen-treated SMYD3-KO mice [11]. In addition, SMYD3 is recruited on the *hTERT* promoter, where it is required for the maintenance of H3K4 trimethylation in CRC and HCC cells. As a result, it supports the occupancy of the trans-activators c-MYC and Sp1, thereby promoting hTERT expression and its telomerase activity, which is essential for replicative immortality [33]. Interestingly, SMYD3 knockdown was shown to induce G2-phase arrest in GC cell lines, along with decreased expression of CDK1 and Cyclin B, which drives entry into mitosis, and higher levels of ATM and its downstream factors p53, CHK2, p21 and phosphorylated-Cdc25C, which contributes to G2 checkpoint control [42]. Furthermore, in HCC and esophageal squamous cell carcinoma, SMYD3 overexpression was associated with the expression of retinoblastoma protein-interacting zinc finger 1 (RIZ1) [22,41], which has a role in the G2/M checkpoint and is downregulated in several types of human cancers [53]. Specifically, high levels of SMYD3 were found to be associated with *RIZ1* promoter hypermethylation, resulting in decreased RIZ1 mRNA expression [22,41].

Taken together, these reports define a critical role for SMYD3 in cell cycle progression. In particular, SMYD3 seems to be involved in S phase transition control and in the subsequent G2 checkpoint, which is a crucial cell cycle “timeout” in which DNA is checked for errors before mitosis can begin. Cancer cells usually ignore cell cycle checkpoints, which can lead to gain-of-function alterations in oncogenes and/or loss-of-function alterations in tumor suppressor genes [54]. In the event of DNA damage, proliferation is stopped and cells activate the DNA repair machinery to correct the error(s) or, when the damage cannot be repaired, they undergo cell death. Indeed, the G2 checkpoint is an essential safeguard mechanism to maintain genomic stability during cell cycle progression and is thus a critical process for cancer initiation and development.

Several studies have investigated cell cycle checkpoint dysfunctions that occur in GI cancers, analyzing their association with tumor aggressiveness and patient outcomes. Similar to SMYD3, other factors that are directly involved in cell cycle regulation, such as cyclins and CDKs, are frequently deregulated in GI tumors and these alterations are correlated with less favorable tumor phenotypes and poor prognosis [55]. Specifically, cyclin D1 has been found overexpressed in most types of GI tumors, with its overexpression being related to poor survival [56,57,58,59,60]; cyclin D2 overexpression has been associated with poor prognosis in CRC and GC [57,60]; cyclin E overexpression has been correlated with aggressiveness and lymph node metastasis in GC [60], and with higher-stage disease in HCC [59]. CDK1/2 overexpression has been reported in CRC and esophageal cancers [58,61], CDK4 overexpression has been described in most types of GI tumors [56,58,59,60] and CDK6 overexpression has been observed in CRC and esophageal cancers [58]. These CDK dysregulations have been frequently associated with poor prognosis [55].

The success of future therapies for cancers with cell cycle vulnerabilities will depend on the identification of the associated cell cycle-related aberrations and the development of selective compounds targeting cell cycle proteins. As such, efforts should be made to gain a better understanding of the role of SMYD3 in cancer cell cycle progression.

### 4.2. Role of SMYD3 in DNA Damage: From Tumorigenesis to Cancer Progression

As referred above, SMYD3 involvement in cancer cell growth has been called into question based on compelling evidence showing that SMYD3 is dispensable for autonomous cell proliferation [28]. On the other hand, the importance of SMYD3 in GI cancer development has been confirmed in vivo by data obtained in mice models in which carcinogenesis was induced chemically. Indeed, SMYD3 was found overexpressed in liver tumors in mice treated with diethylnitrosamine (DEN) as a model for HCC, and in colon tumors in mice treated with dimethylhydrazine/dextran sodium sulfate (DMH/DSS) and APC^Min/+^ mice treated with the carcinogen AOM as a model for CRC [9,11]. Knocking out SMYD3 dramatically reduced the tumor formation capacity induced by these carcinogens, as shown by a decrease in the number and size of tumor foci in the colon and liver compared to wild-type mice [11]. In line with recent observations on SMYD3 involvement in cancer development [28], no spontaneous liver tumor formation was detected in mice constitutively overexpressing SMYD3 in hepatocytes and no differences in tumor foci numbers were observed between wild-type and SMYD3-overexpressing mice after DEN treatment [11]. Remarkably, it has been shown that SMYD3 is required for the compensatory proliferation of cells that escaped apoptosis caused by DEN-induced and DMH/DSS-induced DNA damage [11]. These events, which are involved in carcinogenesis, could be an effect of SMYD3-mediated transcriptional regulation of cancer-related genes, such as *MYC* and *CTNNB1*, and components of the IL6-JAK-STAT3 pathway [11]. This evidence suggests that SMYD3 may play a signal-dependent role in promoting GI cancer formation and development in response to genotoxic stress.

DNA damage response (DDR) is a complex signaling network acting to protect the stability and integrity of the cellular genome [62]. Genotoxic stress activates DDR, which is characterized by a signal transduction cascade that starts with the detection of damaged DNA by specific sensors. These sensors induce cell cycle arrest and stimulate DNA repair by activating specific transducers, which are able to recruit the repair machinery on DNA lesions [63]. Specifically, DNA lesions trigger the activation of various kinases, the most important being the phosphoinositide-3-kinase-related protein kinase (PIKK) family members ATM, ATR and DNA-PKcs. While ATR activation is associated with single-stranded DNA and stalled DNA replication forks, ATM and DNA-PKcs respond mainly to double-strand breaks (DSBs) [64]. These three factors link the DDR machinery to cell cycle checkpoints by arresting cell cycle progression and therefore allowing DNA restoration before cell division. DDR and cell cycle checkpoints act in conjunction to maintain genomic stability, since, on the one hand, impaired DNA repair leads to the introduction of mutations and therefore to potential critical outcomes, and on the other hand cell cycle checkpoint dysfunctions result in the proliferation of DNA-damaged cells [65,66].

Interestingly, we have recently delved into the role of SMYD3 in maintaining genome integrity in a GI cancer context. Since SMYD3 regulates several key cancer-associated proteins through direct interaction, we carried out an in silico peptide screening with the aim of identifying new SMYD3 interactors to better characterize its involvement in cancer progression [45]. We found that SMYD3 directly binds to ATM, CHK2 and BRCA2, which are important sensors and effectors of homologous recombination (HR), a specific signaling cascade that is required for DNA DSB repair. Our results showed that high levels of SMYD3 are required for DNA restoration after the induction of DSBs. Specifically, SMYD3 promotes the formation of HR complexes during DDR by interacting with ATM. This propagates the signal cascade through CHK2 and BRCA2, thereby promoting the recruitment of RAD51 on DNA lesions. Moreover, new findings were obtained based on the identification of a new *SMYD3* genetic variant (p.Arg265His) in a BC high-risk family [45]. This SMYD3-R265H mutant protein, which is predicted to be deleterious and was also found in a dataset of patients with CRC [67,68], shows a stronger interaction with ATM and localizes at DSBs like the wild-type form but is not able to interact with CHK2 and BRCA2. This prevents the recruitment of the DNA repair complex on damage sites, suggesting that this variant may play a dominant-negative role [45].

These new findings reveal an important role for SMYD3 in DNA repair and are supported by another study in which SMYD3 was linked to HR. In this paper, the authors focused on SMYD3-mediated modulation of the expression of genes related to DNA damage response and showed how SMYD3 influences DNA restoration by analyzing long recovery times [69]. In addition, as previously reported, it has been found that SMYD3 genetic ablation upregulates ATM and its downstream signaling cascade, thus suggesting that SMYD3 may influence cell cycle progression through an ATM-dependent mechanism [42].

**Figure 2:**
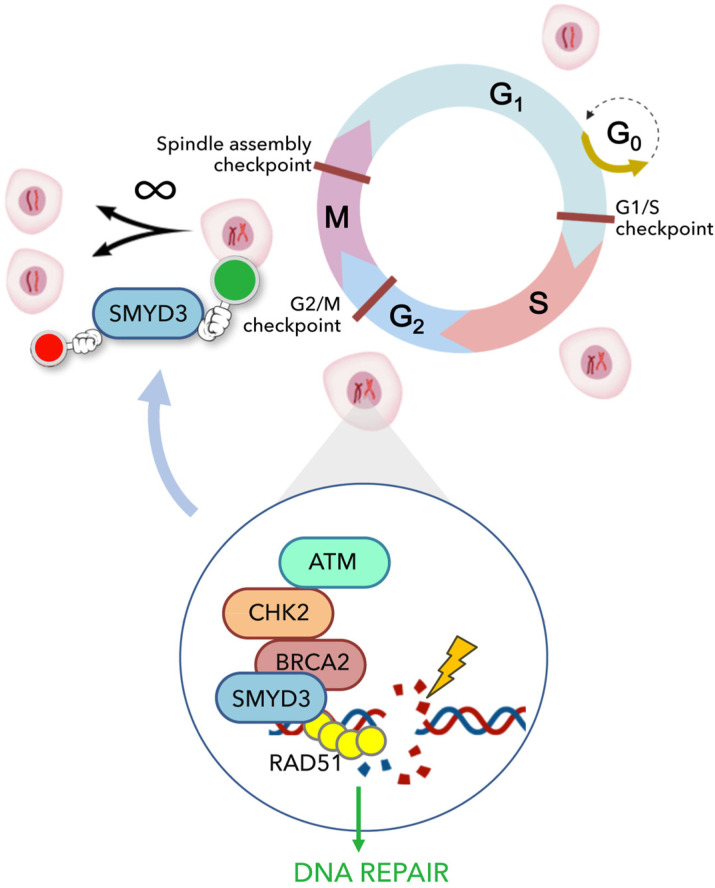
SMYD3 plays an important role in protecting cancer cells and sustaining cancer progression by controlling cell cycle dynamics and promoting DNA repair, thereby allowing unlimited cell proliferation. It is involved in the S/G2 checkpoint, in which newly synthesized DNA is checked for errors before the cell enters mitosis. When DNA damage occurs, SMYD3 arrests the cell cycle in the G2 phase to activate the DNA repair machinery and enable error correction. Following DNA repair, the cell cycle is resumed and cancer cells continue to proliferate.

Taken together, this evidence allows to better define the complex role of SMYD3 in promoting GI cancer cell progression. Firstly, SMYD3 is implicated in cell cycle checkpoint control, through which it induces a high rate of cell division, which is typical of cancer cells. At the same time, it is required for the repair of DNA DSBs caused by genotoxic agents (i.e., neocarzinostatin, chemotherapeutic drugs and other compounds that target the DNA repair machinery), which are highly cytotoxic for cancer cells. This novel SMYD3 function is crucial for sustaining tumor progression since cancer cells need to repair DNA damage to continue to proliferate (Figure 2). These findings reveal that SMYD3 has an important protective function for cancer cells. Indeed, cancer cells display a high incidence of activated oncogenes resulting in uncontrolled pathways that sustain unlimited cell proliferation. This leads to error accumulation and DNA replication stress, which could compromise cell division and cancer progression. In this scenario, SMYD3 overexpression reinforces DNA damage response in cells with intrinsic/genotoxic stress and hence promotes cancer progression, suggesting that it may also alter cell sensitivity to genotoxic cancer therapy.

## 5. Clinical Impact of SMYD3 Inhibition for New Therapeutic Strategies in GI Cancers

### 5.1. Alterations of DNA Damage Checkpoint Factors in GI Cancer Initiation and Development

DNA repair factor aberrations, including both activation and inactivation, occur frequently in human sporadic GI cancers [70]. Recent studies revealed that patients with GI cancers sometimes harbor mutations in BC and OvCa susceptibility genes (i.e., *BRCA1/2*) [71]. A large analysis of DDR pathway genes in tubular GI malignancies identified deleterious alterations in 17% of 17,486 cases. Among 10 predefined DDR genes, *ARID1A* (9.2%) and *ATM* (4.7%) were the most commonly altered ones, followed by *BRCA2* (2.3%), *BRCA1* (1.1%), *CHEK2* (1.0%), *ATR* (0.8%), *CDK12* (0.7%), *PALB2* (0.6%), *CHEK1* (0.1%) and *RAD51* (0.1%) [72]. Moreover, an association between DDR defects and high tumor mutational burden has been found in more than 20% of tubular GI cancer cases [72]. Defects in mismatch repair (MMR) genes and hypermethylation of the *MLH1* gene, which lead to an MMR-deficient phenotype, have been observed in 2–3% and 10% of CRC cases, respectively [73,74,75,76]. Analysis of TCGA datasets for colon (COAD) and rectal (READ) adenocarcinoma identified mutations in several DDR genes, which are considered “putative cancer drivers” as they are not known to drive carcinogenesis but may lead to DNA repair deficiency [76,77].

Intriguingly, a BRCA signature was recently identified in GI cancers, with a significant enrichment of HR gene alterations, including *BRCA1/2* and *PALB2* [78,79]. Another DDR gene that has been found mutated in GI tumors is *ATM* (2–8%), a feature that was not previously reported in TCGA studies [79,80]. *ATM*-mutated CRCs show a significantly longer median overall survival [81]. A prevalence of *ATM* aberrations has been identified in GCs, with patients carrying loss-of-function *ATM* mutations being diagnosed at an earlier age [82]. Of note, as previously referred, we recently found a *SMYD3* genetic variant that was predicted to be deleterious [45], suggesting that, albeit with lower frequency, *SMYD3* may also be involved in genetic susceptibility to inherited cancers.

In addition to genetic mutations, dysregulated expression of DDR genes has also been described in GI cancers [70]. This is particularly true for SMYD3. Indeed, it is becoming increasingly clear that SMYD3 alterations implicated in GI cancers mainly involve its overexpression. As reported above, our analyses to assess *SMYD3* somatic aberrations in CRCs and PCs showed that only a low percentage of tumors harbor *SMYD3* mutations, while higher SMYD3 expression occurs in around one-third of all cases [45]. This is consistent with data obtained in other cancer types [1].

Similar to SMYD3, ATM has been found overexpressed in CRC cells compared to adjacent normal and control tissues, and this feature was correlated with well-differentiated tumors [83]. In addition, increased expression of RAD51 has been observed in PCs, where it was associated with resistance to platinum agents and poor outcome [84], while decreased expression has been described in CRC [85,86]. RAD50 [87] and CHK1 have also been shown to be overexpressed in CRC, with the latter being associated with poor prognosis and resistance to chemotherapy [88].

Further studies are needed to better dissect the link between DDR gene mutations and GI cancer and to establish whether such alterations are causative of these malignancies.

### 5.2. SMYD3 as a Promising Pharmacological Target Involved in DNA Damage Checkpoints in GI Cancers

Cancer cells are strictly dependent on DNA repair for survival and proliferation. Indeed, the DNA repair deficiency that occurs in some cancers results in the activation of alternative repair pathways [66]. These are mediated by PARP1 activity, which plays an important role as a sensor protein recognizing both single-strand breaks and DSBs and recruits DDR factors to the region around DNA lesions, thereby priming the activation of specific DNA repair cascades [89,90]. Recently, studies carried out to devise novel therapeutic strategies for cancer treatment have been focusing on DDR deficiencies with the aim of achieving synthetic lethality, which refers to the induction of cell death through combined deficiencies in the expression or activity of two genes, whereas the perturbation of either gene alone is viable [91,92]. These deficiencies can be the result of genetic mutations, epigenetic alterations or the activity of specific inhibitors. Targeting the rescue DNA repair pathway in cancer cells carrying DDR deficiencies has been recently shown to be an effective strategy for several cancer types, including BRCA1/2-deficient cancers, where the use of PARP inhibitors (PARPi) is a model example of synthetic lethality [91]. The use of PARPi has entered clinical practice following FDA approval for the treatment of OvCas, BCs and PCs harboring defects in HR genes [93], which define a *BRCA*ness phenotype. The first PARPi, Olaparib, was approved for *BRCA*-mutated OvCa in 2014 [94]. It has subsequently been included in clinical trials for various types of GI cancers, including esophageal cancer, recurrent or metastatic GC, advanced PC and CRC, often in combination with radio- and chemotherapy [70], since previous studies had shown a synergistic response to the combined treatment with PARPi and specific chemotherapeutics in GI cancers [76,95,96].

Currently, pharmaceutical companies are also directing their attention to other DDR and cell cycle checkpoint factors, including CHK1/2, ATM, ATR, DNA-PKcs and RAD51, in order to develop cancer treatments to be used either alone or in combination with other anticancer drugs [70]. In addition, recent studies in preclinical models have shown the potential of the pharmacological inhibition of a DDR factor in a setting where another DDR factor is functionally defective [96].

**Figure 3:**
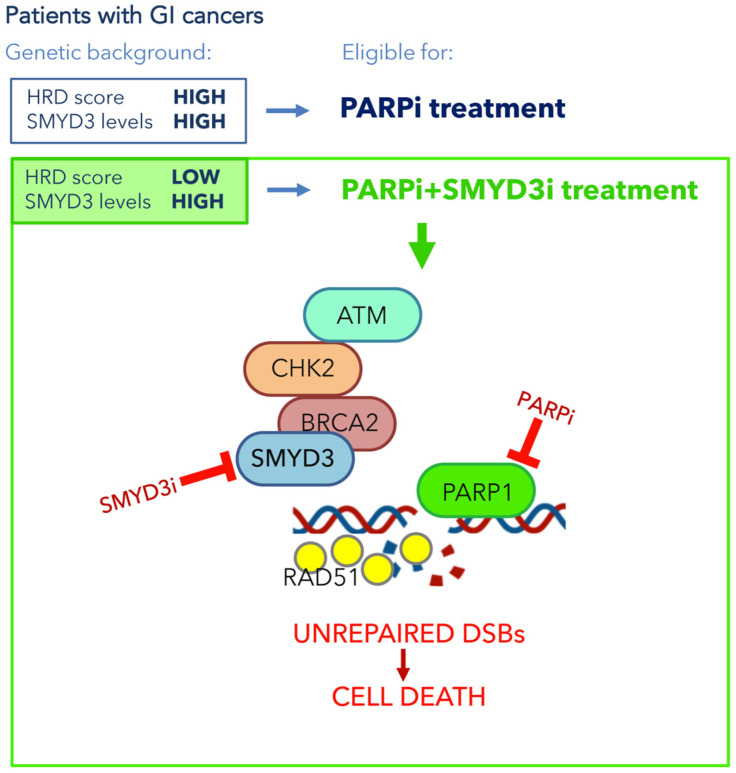
SMYD3 has been found highly overexpressed in most of GI cancers. PARP inhibition is a promising therapeutic strategy for tumors with a high HRD score (HR-deficient). New evidence supports the potential of a novel therapeutic strategy for GI cancers with a low HRD score (HR-proficient) and high levels of SMYD3. This strategy is based on a synthetic lethality approach consisting of the combined treatment with SMYD3i and PARPi. This would alter GI cancer cell ability to restore DNA damage and therefore lead to cell death. GI: gastrointestinal; SMYD3i: SMYD3 inhibitor; PARPi: PARP inhibitor; DSBs: double-strand breaks.

Previous data from our group suggested the possible activation of compensatory DNA repair signals after inhibition of SMYD3 to impair HR repair. Since the activation of alternative DNA repair mechanisms requires PARP1 activity, we hypothesized that combined inhibition of SMYD3 and PARP1 with specific compounds could act as a synthetic lethality strategy. Specifically, we selected a tumor subset that is HR-proficient, and therefore addicted to high levels of SMYD3, as a candidate for this new therapeutic strategy based on the impairment of HR repair response with a specific SMYD3i to make the tumor sensitive to PARPi. Based on our hypothesis, the combined treatment would alter cancer cell ability to restore DNA damage and therefore cause cell death. Our results confirmed its potential, with the combined use of SMYD3i and PARPi showing a cytotoxic effect in CRC and PC cell lines [45]. Based on these findings, a synthetic lethality approach may be extended to a fraction of human tumors determined to be eligible for this therapeutic strategy. Eligibility could be assessed by evaluating a recently defined biomarker named HR deficiency (HRD) score, which determines the HR repair response status by analyzing specific standardized parameters [77]. Tumors with a low HRD score (meaning they are HR repair proficient) and high SMYD3 expression are expected to be the best candidates for the combined treatment (Figure 3). Based on an analysis of the PanCanAtlas dataset, we found that 41.2% of CRC tumors (from the COAD-READ dataset) with high SMYD3 mRNA levels have a low HRD score. Intriguingly, we also found that CRCs displayed mutual exclusivity of SMYD3 overexpression and genetic alterations of major HR genes that were previously shown to be correlated with a higher HRD score [45]. In the same study, we extended this analysis to another type of GI cancer by assessing a PC tumor dataset (PAAD). Our findings revealed that about 11% of total CRCs and PCs could be eligible for the combined treatment with SMYD3i and PARPi [45].

Altogether, this evidence supports the potential of a novel therapeutic strategy using a combination of SMYD3i and PARPi for HR-proficient tumors expressing high levels of SMYD3 (Figure 3). Based on our data on CRC and PC cell lines and on patient information from the above-mentioned datasets, this approach may be particularly effective in GI cancers.

## 6. Conclusions and Future Directions

SMYD3 mediates the progression of several cancer types by regulating oncogenic mechanisms and signaling pathways in different ways. Here, we focused on SMYD3 involvement in tumors related to the GI compartment, where its altered expression has been found linked to cancer initiation, progression and aggressiveness. SMYD3 can promote cancer by co-regulating the activation of major cancer-related pathways and can act as a critical driver in tumorigenesis. As for its role in cancer progression, SMYD3 has been described as a core promoter of cell cycle regulation that is involved in phase transition and allows cancer cells to bypass signals of cell cycle arrest, thereby contributing to uncontrolled proliferation. In addition, it has a protective role against genotoxic stress, which is critical for cancer development. SMYD3 contributes to the restoration of damaged DNA in cancer cells and therefore enables unperturbed cell division. Thus, SMYD3 appears as a genetic guardian of DNA damage checkpoint dynamics, driving cell cycle phase transition and promoting genomic protection of cancer cells.

Based on these findings, SMYD3 is emerging as an important target for drug discovery. Further studies are needed not only to gain a full comprehension of SMYD3-mediated mechanisms promoting cancer progression but also to gather stronger evidence in support of the effectiveness of novel therapeutic strategies based on the use of SMYD3i in specific patient subsets. Moreover, future studies will have to focus on the design of novel inhibitors suitable for cancer patients, with the aim of making SMYD3 a druggable target in clinical practice. Indeed, combining currently used DNA-damaging drugs with compounds that target DNA damage checkpoints can lead cancer cells to overcome repair mechanisms and cell cycle arrest, thereby undergoing cell death. In this light, a thorough understanding of the effects of SMYD3 inhibition may help to devise more selective and efficient pharmacological interventions for GI cancer patients in the clinical setting. In particular, it may permit the improvement of current therapies by combining them with SMYD3i to sensitize GI cancers expressing high levels of SMYD3.
